# 不同胶原酶对慢性移植物抗宿主病小鼠唾液腺免疫细胞分析的影响

**DOI:** 10.3760/cma.j.cn121090-20260122-00048

**Published:** 2026-03

**Authors:** 紫祎 胡, 清晓 宋, 世杰 杨, 锦 魏, 曦 张

**Affiliations:** 1 川北医学院临床医学院，南充 637000 Department of Clinical Medicine, North Sichuan Medical College, Nanchong 637000, China; 2 川北医学院附属医院血液内科，南充 637002 Department of Hematology, Affiliated Hospital of North Sichuan Medical College, Nanchong 637002, China; 3 陆军军医大学第二附属医院血液病医学中心，血液生态与智慧细胞科学创新中心，全军临床重点专科，创伤与化学中毒国家重点实验室，血液病与微环境重庆市重点实验室，重庆 400037 Medical Center of Hematology, Xinqiao Hospital of Army Medical University, Blood Ecology and Smart Cell Science Innovation Center, State Key Laboratory of Trauma and Chemical Poisoning, Chongqing Key Laboratory of Hematology and Microenvironment, Chongqing 400037, China; 4 金凤实验室，重庆 401329 Jinfeng Laboratory, Chongqing 401329, China

**Keywords:** 移植物抗宿主病, 唾液腺，胶原酶Ⅱ, 胶原酶D, 免疫细胞, Graft versus host disease, Salivary glands, Collagenase Ⅱ, Collagenase D, Immune cells

## Abstract

**目的:**

初步探讨不同胶原酶对慢性移植物抗宿主病（cGVHD）小鼠唾液腺免疫细胞分析的影响。

**方法:**

构建主要组织相容性复合体不相合小鼠模型（骨髓：5×10^6^个细胞；脾：0.25×10^6^个细胞），包括无GVHD组（BALB/c小鼠供BALB/c小鼠，15只）和cGVHD组（C57BL/6小鼠供BALB/c小鼠，15只），移植后观察小鼠体重、症状评分和生存率。在移植后第45天，收集各组小鼠的唾液腺（6～12只），进行HE和Masson三色染色评估损伤及纤维化程度；通过流式细胞术分析，比较无胶原酶消化、Ⅱ型胶原酶消化和D型胶原酶消化对免疫细胞表型的影响。

**结果:**

与无GVHD组相比，cGVHD组小鼠移植后体重下降［移植后第60天初始体重占比:（66.77±11.72）％对（101.20±4.19）％，*P*<0.0001］，症状评分高（2.11±0.59对0±0，*P*<0.0001），存活率降低［移植后第60天存活率：50％对100％，*P*＝0.0011］。cGVHD小鼠唾液腺表现为腺泡结构萎缩，细胞浸润（病理评分：3.67±0.52对2.00±0，*P*<0.0001）和明显纤维化［胶原纤维面积占比：（14.54±4.05）％对（4.11±0.87）％，*P*＝0.0001］。使用胶原酶消化的方法显著增加了单个核细胞的总数和活率（*P*值均<0.05），其中含Ⅱ型胶原酶方案获得的单个核细胞数量最高（*P*<0.01）。但是Ⅱ型胶原酶消化降低了CD4^+^ T细胞比例［（5.64±2.05）％对（31.98±12.23）％，*P*＝0.0052］，而D型胶原酶对CD4^+^ T细胞无明显影响［（31.51±16.37）％对（31.98±12.23）％，*P*＝0.9973］。cGVHD组中，含D型胶原酶方案能够获得更多CD4^+^ T细胞而含Ⅱ型胶原酶方案能够获得更多CD8^+^T细胞（*P*值均<0.05）。NK细胞数量在两种胶原酶处理下均显著增加（*P*值均<0.05）。在髓系细胞中，Ⅱ型胶原酶降低Ly6C^low^CD11b^+^Ly6G^+^中性粒细胞比例［（0.05±0.01）％对（0.34±0.20）％，*P*＝0.0171］，D型胶原酶无影响［（0.49±0.19）％对（0.34±0.20）％，*P*＝0.2800］。在cGVHD组中，D型胶原酶处理后获得的CD11b^+^Ly6C^+^Ly6G^-^单核细胞数量高于Ⅱ型胶原酶［（0.06±0.05）×10^5^对（0.01±0）×10^5^，*P*＝0.0384］，而Ⅱ型胶原酶处理后获得的巨噬细胞数量明显高于D型胶原酶［（1.42±0.56）×10^5^对（0.57±0.36）×10^5^，*P*<0.0001］。

**结论:**

使用胶原酶消化显著增加了单个核细胞的数量和活率。其中含Ⅱ型胶原酶方案对分离CD8^+^ T细胞、NK细胞和巨噬细胞最优；D型胶原酶则更有效地富集CD4^+^T细胞、中性粒细胞与单核细胞。

## Abstract (en)

**Objective:**

To preliminarily explore the influence of different collagenases on the analysis of immune cells in salivary glands of mice with chronic graft-versus-host disease（cGVHD）.

**Methods:**

A major histocompatibility complex mismatch model was established（bone marrow: 5×10^6^ cells; spleen: 0.25×10^6^ cells）, including the non-graft-versus-host disease（GVHD）control group（BALB/c to BALB/c, *n*＝15）and cGVHD group（C57BL/6c to BALB/c, *n*＝15）. Body weight, clinical scores, and survival rates of the mice were observed after transplantation. On day 45 after transplantation, salivary glands were collected（*n*＝6–12）, and hematoxylin and eosin and Masson trichrome staining were performed to assess the degree of injury and fibrosis. Flow cytometry analysis was conducted to compare immune cell populations and phenotypes among samples without collagenase digestion, with collagenase Ⅱ digestion, and with collagenase D digestion.

**Results:**

Compared with the non-GVHD group, mice in the cGVHD group showed significant weight loss［original body weight percentage on day 60 after transplantation:（66.77±11.72）％ *vs*（101.20±4.19）％, *P*<0.0001］, higher symptom scores（2.11±0.59 *vs* 0±0, *P*<0.0001）, and significantly lower survival rates（survival rate on day 60 after transplantation: 50％ *vs* 100％, *P*＝0.0011）. The salivary glands of cGVHD mice showed acinar atrophy, cellular infiltration（3.67±0.52 *vs* 2.00±0, *P*<0.0001）, and fibrosis［（14.54±4.05）％ *vs*（4.11±0.87）％, *P*＝0.0001］. Collagenase digestion significantly increased the yield and viability of mononuclear cells（*P*<0.05）. Among the tested protocols, digestion with collagenase Ⅱ resulted in the greatest recovery of mononuclear cells（*P*<0.01）. However, collagenaseⅡdigestion significantly reduced the proportion of CD4^+^ T cells［（5.64±2.05）％ *vs*（31.98±12.23）％, *P*＝0.0052］, whereas collagenase D had no significant effect on CD4^+^ T cells［（31.51±16.37）％ *vs*（31.98±12.23）％, *P*＝0.9973］. In the cGVHD group, the method containing collagenase D recovered more CD4^+^ T cells, whereas the method containing collagenase Ⅱ recovered more CD8^+^ T cells（all *P* value<0.05）. In addition, the number of natural killer（NK）cells was significantly increased in both groups after collagenase digestion（all *P* value<0.05）. Among myeloid cells, collagenaseⅡdigestion significantly reduced the proportion of Ly6C^low^CD11b^+^Ly6G^+^ neutrophils［（0.05±0.01）％ *vs*（0.34±0.20）％, *P*＝0.0171］, whereas collagenase D had no significant effect［（0.49±0.19）％ *vs*（0.34±0.20）％, *P*＝0.2800］. In cGVHD tissues, the number of CD11b^+^Ly6C^+^Ly6G^-^ monocytes rcovered with collagenase D digestion was significantly higher than that recovered with collagenase Ⅱ digestion［（0.06±0.05）×10^5^ *vs*（0.01±0）×10^5^, *P*＝0.0384］, whereas the number of macrophages rcovered with collagenase Ⅱ digestion was significantly higher than that recovered with collagenase D digestion［（1.42±0.56）×10^5^ *vs*（0.57±0.36）×10^5^, *P*<0.0001］.

**Conclusion:**

Collagenase digestion significantly increased the number and viability of mononuclear cells. The method containing collagenase Ⅱ yielded the highest number of mononuclear cells as well as CD8^+^ T cells, NK cells, and macrophages, whereas collagenase D was more suitable for recovering CD4^+^ T cells, neutrophils, and monocytes.

异基因造血干细胞移植（allo-HSCT）是治疗血液系统恶性疾病的主要手段之一，主要依靠供者来源的免疫细胞介导的移植物抗白血病效应。然而，这些免疫细胞也可能攻击患者的器官组织，导致移植物抗宿主病（GVHD）的发生[1]。慢性GVHD（cGVHD）的发生率为30％～70％，可影响多种器官，以眼、皮肤、口腔和肺最为常见，严重影响了患者的生存质量，甚至危及生命[2]。cGVHD发病机制复杂，胸腺功能的受损导致多种免疫细胞的活化及扩增，包括T细胞、B细胞和巨噬细胞；在cGVHD晚期，成纤维细胞活化导致组织修复异常和纤维化[3]。目前，基于患者外周血或骨髓样本的研究通常难以准确反映靶器官的病理过程，因此，利用动物模型深入探索cGVHD的发病机制是必要且常用的研究策略。

当前的动物模型研究主要集中在cGVHD靶器官（皮肤、肝脏、肺和肠道）等[4]–[7]，而对口腔cGVHD的研究相对较少。口腔cGVHD的发病率为30％～70％，患者常伴有唾液腺损伤，表现为唾液分泌明显减少，口腔黏膜破损以及合并细菌、真菌感染[8]–[9]。唾液腺的损伤在组织病理上表现为T细胞和巨噬细胞浸润腺体及导管周围[10]–[11]。然而，免疫细胞如何相互作用并导致唾液腺损伤的具体机制尚未完全阐明。因此，亟需对唾液腺组织的免疫细胞进行分离和进一步分析。

组织分离、酶消化和机械解离是获得高质量单细胞悬液的关键步骤[12]。其中，酶消化在降解胶原蛋白、细胞外基质、黏附分子及细胞间连接方面至关重要。然而，研究表明，胶原酶的使用可能会导致免疫细胞表面抗原的降解[13]–[14]，进而引起最终获取的免疫细胞组成成分出现偏差，无法反映组织微环境的客观现象。目前获取唾液腺单个核细胞的消化方案主要采用含Ⅱ型胶原酶和（或）D型胶原酶的方案，其分离效果在不同疾病表型间存在差异[15]–[18]。本研究选取CD45、CD3、CD4、CD8、CD11b及主要组织相容性复合体（MHC）-Ⅱ等作为主要分析的表面抗原，分别代表唾液腺组织中免疫细胞总体组成、T细胞群体以及髓系细胞及其抗原呈递功能，是评估局部免疫微环境的基础性指标。本研究旨在比较两类胶原酶从小鼠唾液腺中分离上述类型免疫细胞的效果，为研究口腔cGVHD的发病机制奠定实验技术基础。

## 材料与方法

1. 实验动物：8～12周龄的无特殊病原体（SPF）级雄性C57BL/6（H-2b）小鼠10只和BALB/c（H-2d）小鼠70只，均购自北京维通利华实验动物技术有限公司。所有小鼠均饲养于陆军军医大学实验动物中心SPF级动物培养室，且所有实验方案均经陆军军医大学新桥医院机构动物保护与使用委员会批准（批件号：AMUWEC20245225），采用二氧化碳（CO₂）吸入法处死小鼠。

2. cGVHD动物模型的构建与评估：构建MHC不相合模型，供体小鼠为C57BL/6，受体小鼠为BALB/c。设无GVHD为对照组（BALB/c供BALB/c）以及cGVHD组（C57BL/6供BALB/c），每组各15只。8～12周龄的雄性BALB/c受体小鼠接受了750 cGy的辐照（X射线）处理。在第2次辐射后6～8 h，分离供体小鼠的单个核细胞（骨髓：5×10^6^个细胞；脾：0.25×10^6^个细胞），通过尾静脉注射到受体小鼠体内。移植后观察小鼠的体重变化（初始体重占比：观察体重除以移植当天的初始体重×100％），弓背、腹泻、脱毛等cGVHD症状以及统计存活率，根据症状的轻重及持续时间评估cGVHD症状的分值，具体评分标准见参考文献[19]。

3. 苏木精-伊红（HE）染色以及cGVHD评分标准：组织经甲醛溶液固定、石蜡包埋、切片及HE染色后，用Olympus光学显微镜拍摄图像。唾液腺cGVHD具体评分标准[20]：1分：可见1至5个单核细胞病灶（每个病灶超过20个细胞）；2分：可见超过5个单核细胞病灶，但无显著实质破坏；3分：可见多个融合病灶，伴有实质组织的中度变性；4分：腺体内有大量单核细胞浸润，并且实质组织遭到严重破坏。

4. Masson三色染色：组织切片常规脱蜡至水，将脱好水的切片浸泡于Bouin固定液或Zenker固定液过夜，次日流水冲洗干净；Harris苏木素或铁苏木素染色5～10 min，流水冲洗；用0.8％～1％盐酸乙醇分化数秒，流水冲洗数分钟；碳酸锂返蓝数秒后流水冲洗；丽春红酸性品红染液染5～10 min，流水稍冲洗；磷钼酸溶液处理约5 min后直接使用苯胺蓝染液复染5 min；1％冰醋酸处理1 min；95％酒精脱水多次；随后依次用无水酒精脱水、二甲苯透明化及中性树胶封固。用Olympus光学显微镜拍摄图像，胶原纤维呈蓝色或绿色；胞质、肌纤维和红细胞呈红色；细胞核呈蓝褐色。定量方法见文献[21]。

5. 消化液制备：每个样本配制500 µl的消化液，无胶原酶即对照组消化液成分为0.1 mg/ml DNAse Ⅰ（瑞士Roche公司）、0.8 mg/ml分散酶-Ⅱ（瑞士Roche公司）、10 mmol/L Hepes（美国Sigma-Aldrich公司）以及含0.1 mol/L氯化钙的RPMI 1640培养基（上海VivaCell公司）+2％胎牛血清（美国Sigma-Aldrich公司）混合液。实验组除上述成分外，再分别加入100 mg/ml胶原酶-Ⅱ（美国Sigma-Aldrich公司）或100 mg/ml胶原酶-D（瑞士Roche公司）。

6. 单细胞悬液制备：唾液腺分离后，置于含有2％胎牛血清的RPMI 1640培养基中，用外科剪切碎；将组织碎片置于24孔板，加入消化液后在100 r/min、37 °C的振荡培养箱中孵育45 min；随后研磨组织、过滤，4 °C，400×*g*，离心5 min；离心后收集细胞沉淀物，冰上用1×红细胞裂解缓冲液（北京索莱宝公司）裂解5 min，4 °C，400×*g*，离心5 min；离心后收集沉淀，使用40％及70％ percoll液（美国Cytiva公司）进行梯度离心；收集中间白膜层后用培养基重悬，获得单细胞悬液。

7. 流式细胞术分析：CD45（货号：417-0454-82，美国ThermoFisher Bioscience公司）标记免疫细胞，TCR-β（货号：109220，美国Biolegend公司）、CD4（货号：25-0042-82，美国ThermoFisher Bioscience公司）、CD8（货号：553030，美国BD公司）标记T细胞，B220（货号：47-0452-82，美国ThermoFisher Bioscience公司）标记B细胞。在分选T细胞和B细胞后，用NKP46（货号：12-3351-82，美国ThermoFisher Bioscience公司）标记NK细胞，CD11b（货号：101228，美国Biolegend公司）标记髓系细胞。在髓系细胞中用Ly6C（货号：128041，美国Biolegend公司）标记单核细胞，Ly6G（货号：127641，美国Biolegend公司）标记中性粒细胞；在分选单核细胞和中性粒细胞后用F4/80（货号：743281，美国BD公司）标记巨噬细胞、CD11c（货号：117347，美国Biolegend公司）标记树突状细胞，使用CD86（货号：17-0862-82，美国ThermoFisher Bioscience公司）、CD206（货号：12-2069-42，美国ThermoFisher Bioscience公司）、I-A/I-E（货号：107631，美国Biolegend公司）进行平均荧光强度（MFI）值检测。

CD16/32（美国BD公司），LIVE/DEAD、Foxp3/转录因子流式固定破膜缓冲液和500×细胞刺激剂购自美国ThermoFisher Bioscience公司。染色方案具体参照抗体说明书。流式细胞术分析使用BDLSRFortessa（美国BD公司）进行，所得数据使用FlowJo软件V11（美国BD公司）进行分析。

8. 统计学处理：采用Graphpad prism 9.5软件进行统计分析与作图。计量资料以“*x*±*s*”表示。存活率比较采用Log-rank检验。两组间比较采用非配对*t*检验，3组及以上比较采用One-way ANOVA分析，动物实验生物学重复至少3次。*P*<0.05为差异具有统计学意义。

## 结果

1. 构建cGVHD模型：cGVHD组小鼠的症状评分高于无GVHD组（2.11±0.59对0±0，*P*<0.0001，图1A），表现为cGVHD组小鼠在移植后逐渐出现弓背、尾部结痂、腹泻、脱毛等症状且逐渐加重；无GVHD组在移植后1周内有轻度弓背，1周后逐渐消失。cGVHD组小鼠体重持续下降且不能恢复，无GVHD组小鼠仅在移植后1周内出现体重轻度下降，随后体重逐渐恢复［移植后第60天初始体重占比：（66.77±11.72）％对（101.20±4.19）％，*P*<0.0001，图1B］。cGVHD组小鼠从移植后第30天开始陆续出现死亡，移植后第60天存活率为50％，而无cGVHD组小鼠全部存活（*P*＝0.0011）。为评估唾液腺损伤情况，移植后第45天收集唾液腺进行HE染色及Masson三色染色。与无cGVHD组相比，cGVHD组唾液腺腺泡萎缩，细胞在腺泡及导管周围浸润增加，病理评分（3.67±0.52对2.00±0，*P*<0.0001，图1C）及胶原纤维面积占比增高［（14.54±4.05）％对（4.11±0.87）％，*P*＝0.0001，图1D］。以上结果提示成功构建cGVHD小鼠模型，口腔cGVHD的主要组织病理表现为唾液腺组织纤维化。

**图1:**
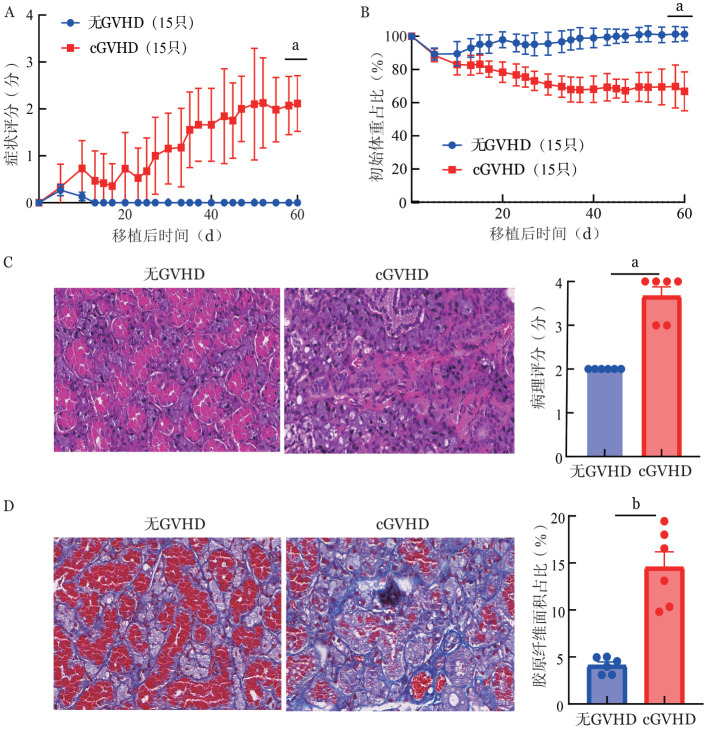
慢性移植物抗宿主病（cGVHD）小鼠模型构建及唾液腺cGVHD特征 **A** 小鼠cGVHD症状评分曲线，cGVHD组（15只）、无GVHD组（15只）；**B** 小鼠体重占移植当天初始体重的百分比曲线，cGVHD组（15只）、无GVHD组（15只）；**C** 小鼠唾液腺HE染色（×100），cGVHD组（6只）、无GVHD组（6只）；**D** 小鼠唾液腺Masson三色染色（×100），cGVHD组（6只）、无GVHD组（6只） **注** GVHD：移植物抗宿主病；^a^*P*<0.0001；^b^*P*<0.001

2. 不同胶原酶消化对细胞活率及数量的影响：为评估不同类型胶原酶消化的方法对唾液腺细胞数量及表型的影响，我们在移植后第45天分离小鼠唾液腺，比较了以下3种消化方案：无胶原酶方案、含Ⅱ型胶原酶方案、含D型胶原酶方案。在解离消化、研磨过滤、红细胞裂解和密度梯度离心分离后获得单个核细胞，进行流式细胞术检测（表1）。

**表1:** 不同胶原酶对慢性移植物抗宿主病（cGVHD）小鼠唾液腺免疫细胞的影响（*x*±*s*）

细胞类型		cGVHD组						无GVHD组					
		无胶原酶方案	含Ⅱ型胶原酶方案	含D型胶原酶方案	*P*1值	*P*2值	*P*3值	无胶原酶方案	含Ⅱ型胶原酶方案	含D型胶原酶方案	*P*1值	*P*2值	*P*3值
CD45^+^细胞	细胞活率（％）	70.83±4.36	95.15±1.24	83.00±5.23	<0.0001	0.0003	0.0003	74.22±2.22	83.47±3.64	83.47±8.41	0.0257	0.0257	>0.9999
	数量（×10^5^）	2.21±1.32	15.96±5.06	7.31±1.79	<0.0001	0.0099	<0.0001	4.02±1.13	14.00±3.00	9.74±1.63	<0.0001	<0.0001	0.0018
CD4^+^ T细胞	比例（％）	31.98±12.23	5.64±2.05	31.51±16.37	0.0052	0.9973	0.0036	0.62±0.41	0.41±0.31	0.35±0.43	0.6041	0.4485	0.9644
	数量（×10^5^）	0.25±0.23	0.05±0.02	2.70±1.48	0.9353	0.0005	0.0002	0.01±0.01	0.01±0.01	0.01±0.01	0.7680	0.9229	0.5626
CD8^+^ T细胞	比例（％）	51.53±4.66	48.25±9.22	60.98±13.78	0.8512	0.2450	0.0909	38.00±12.86	34.73±1.84	34.63±3.09	0.7540	0.7411	0.9997
	数量（×10^5^）	0.55±0.43	3.32±1.45	1.92±1.92	0.0128	0.2280	0.2193	0.30±0.18	0.82±0.36	0.71±0.19	0.0050	0.0270	0.7137
B细胞	活细胞占比（％）	0.92±0.41	0.40±0.18	0.53±0.14	0.0129	0.0625	0.6996	1.06±0.26	0.76±0.53	0.87±0.21	0.1741	0.5385	0.8354
	数量（×10^5^）	0.02±0.01	0.04±0.02	0.03±0.02	0.4778	0.9501	0.6588	0.03±0.01	0.32±0.20	0.09±0.03	<0.0001	0.6188	0.0018
NK细胞	活细胞占比（％）	0.72±0.41	1.45±0.38	1.51±0.59	0.0432	0.0278	0.9719	3.44±0.50	3.32±0.57	3.97±0.57	0.9276	0.2443	0.1341
	数量（×10^5^）	0.02±0.02	0.12±0.03	0.07±0.02	<0.0001	0.0097	0.0133	0.11±0.05	0.83±0.14	0.47±0.15	<0.0001	0.0003	0.0003
中性粒细胞	活细胞占比（％）	0.34±0.20	0.05±0.01	0.49±0.19	0.0171	0.2800	0.0007	0.05±0.05	0.03±0.01	0.04±0.02	0.5588	0.7424	0.9499
	数量（×10^5^）	0.01±0.01	0±0	0.05±0.02	0.5997	0.0034	0.0005	0.01±0.01	0±0	0.01±0	0.6696	0.8575	0.9405
单核细胞	活细胞占比（％）	0.39±0.17	0.16±0.04	0.53±0.27	0.1135	0.3680	0.0078	0.78±0.29	0.56±0.10	0.69±0.19	0.2042	0.7483	0.5496
	数量（×10^5^）	0.02±0.01	0.01±0	0.06±0.05	0.9876	0.0512	0.0384	0.02±0.01	0.08±0.01	0.07±0.02	<0.0001	0.0009	0.4912
巨噬细胞	活细胞占比（％）	4.82±2.10	7.24±1.86	5.44±1.83	0.0795	0.8420	0.2255	16.58±3.16	21.13±2.47	12.26±2.85	0.0357	0.0464	0.0002
	数量（×10^5^）	0.13±0.08	1.42±0.56	0.57±0.36	<0.0001	0.1794	0.0037	0.56±0.18	3.05±0.90	1.25±0.38	<0.0001	0.1231	0.0002

**注** GVHD：移植物抗宿主病；*P*1：无胶原酶方案对含Ⅱ型胶原酶方案；*P*2：无胶原酶方案对含D型胶原酶方案；*P*3：含Ⅱ型胶原酶方案对含D型胶原酶方案

首先比较CD45^+^细胞的细胞活率和数量。与无胶原酶处理相比，胶原酶处理后的细胞活率和数量均增加（*P*值均<0.05）。其中，含Ⅱ型胶原酶方案获得的细胞数量高于含D型胶原酶方案［cGVHD组：（15.96±5.06）×10^5^对（7.31±1.79）×10^5^，*P*<0.0001；无GVHD组：（14.00±3.00）×10^5^对（9.74±1.63）×10^5^，*P*＝0.0018］。上述结果表明，使用胶原酶消化唾液腺有助于增加获得的单细胞的细胞活率及数量且Ⅱ型胶原酶优于D型胶原酶。

3. 不同胶原酶对T细胞的影响：如表1所示，在cGVHD组中，与无胶原酶方案［（31.98±12.23）％］相比，使用含Ⅱ型胶原酶方案消化后，得到的CD4^+^T细胞比例显著降低［（5.64±2.05）％，*P*＝0.0052］，而D型胶原酶消化不影响CD4^+^T细胞比例［（31.51±16.37）％，*P*>0.05］且获得的细胞数量增加［（2.70±1.48）×10^5^对（0.25±0.23）×10^5^，*P*＝0.0005］；在无GVHD组中，3种消化方案获得的CD4^+^T细胞比例及数量差异均无统计学意义（*P*值均>0.05）。与无胶原酶相比，胶原酶对CD8^+^T细胞比例无影响（*P*值均>0.05），Ⅱ型胶原酶处理后获得的CD8^+^T细胞数量在cGVHD组增加［（3.32±1.45）×10^5^对（0.55±0.43）×10^5^，*P*＝0.0128］，而在无GVHD组，两种胶原酶处理后的CD8^+^T细胞数量均增加（*P*值均<0.05）。上述结果提示，对唾液腺T细胞进行分析时，应该选用含有D型胶原酶方案，含有Ⅱ型胶原酶方案会降低CD4^+^T细胞比例，导致获得的CD4^+^T细胞的比例及数量假性减少。

4. 不同胶原酶对B细胞和NK细胞的影响：如表1所示，在cGVHD组中唾液腺经胶原酶处理后B细胞数量较无胶原酶处理差异无统计学意义（*P*值均>0.05）；无cGVHD组中与未使用胶原酶相比［（0.03±0.01）×10^5^］，唾液腺经Ⅱ型胶原酶消化后获得的B细胞数量增加［（0.32±0.20）×10^5^，*P*<0.0001］，而D型胶原酶处理差异无统计学意义［（0.09±0.03）×10^5^，*P*＝0.6188］。与无胶原酶方案相比，无论是无GVHD组还是cGVHD组，胶原酶消化后的NK细胞的数量均增加且Ⅱ型胶原酶效果更好（*P*值均<0.05）。上述结果表明，对唾液腺B细胞和NK细胞进行分析时，应该选用含Ⅱ型胶原酶方案。

5. 胶原酶对唾液腺中性粒细胞和单核细胞表型的影响：在cGVHD组中，与无胶原酶方案相比［（0.34±0.20）％］，Ⅱ型胶原酶消化降低了Ly6C^low^CD11b^+^Ly6G^+^中性粒细胞的比例［（0.05±0.01）％，*P*＝0.0171］，而D型胶原酶对该群细胞无明显影响［（0.49±0.19）％，*P*＝0.2800］。在cGVHD组中，与无胶原酶方案相比，两种胶原酶处理后CD11b^+^Ly6C^+^Ly6G^-^单核细胞的数量均未增加（*P*值均>0.05），但D型胶原酶消化获得的单核细胞比例及数量均高于Ⅱ型胶原酶组［（0.53±0.27）％对（0.16±0.04）％，*P*＝0.0078；（0.06±0.05）×10^5^对（0.01±0）×10^5^，*P*＝0.0384）］。而在无cGVHD组中，与无胶原酶方案相比，Ⅱ型及D型胶原酶均增加该群细胞的数量［（0.08±0.01）×10^5^、（0.07±0.02）×10^5^对（0.02±0.01）×10^5^，*P*<0.001］，但其比例未发生明显变化且两种胶原酶间差异无统计学意义（*P*值均>0.05）（表1）。上述结果提示，对唾液腺中性粒细胞和单核细胞进行分析时，应选用含D型胶原酶方案，含Ⅱ型胶原酶方案会导致获得的Ly6C^low^CD11b^+^Ly6G^+^中性粒细胞的比例及数量减少。

6. 不同胶原酶消化对唾液腺巨噬细胞数量及表型的影响：与无胶原酶方案相比，在cGVHD组及无GVHD组中，Ⅱ型胶原酶消化后最终获得的CD11c^+^CD11b^+^F4/80^+^巨噬细胞数量增加［cGVHD组：（1.42±0.56）×10^5^对（0.13±0.08）×10^5^，*P*<0.0001；无GVHD组：（3.05±0.90）×10^5^对（0.56±0.18）×10^5^，*P*<0.0001］，而D型胶原酶消化后无影响（*P*值均>0.05，表1）。进一步对巨噬细胞亚群进行分析（表2），无GVHD组中，Ⅱ型胶原酶处理后上调巨噬细胞CD86的表达水平（1 752.00±152.40对1 221.00±68.25，*P*<0.0001），在cGVHD组中差异无统计学意义（*P*>0.05）。在cGVHD组及无GVHD组中，Ⅱ型胶原酶处理后巨噬细胞CD206的表达水平均上调（cGVHD组：149.30±37.02对44.68±29.70，*P*＝0.0002；无GVHD组：230.00±15.80对21.94±13.48，*P*<0.0001）。而D型胶原酶处理未影响CD86表达水平（*P*>0.05），但在cGVHD组中降低CD206表达水平（−56.84±67.22对44.68±29.7，*P*＝0.0003）。在无GVHD组及cGVHD组中，两种胶原酶对巨噬细胞MHC-Ⅱ的表达水平均无明显影响（*P*值均>0.05）。上述结果表明，对唾液腺巨噬细胞进行分析时，应选用含Ⅱ型胶原酶方案，Ⅱ型胶原酶消化后有助于富集CD206^+^的M2型巨噬细胞。

**表2:** 不同胶原酶对慢性移植物抗宿主病（cGVHD）小鼠唾液腺巨噬细胞表型的影响（平均荧光强度，*x*±*s*）

细胞分子类型	cGVHD组						无GVHD组					
	无胶原酶方案	含Ⅱ型胶原酶方案	含D型胶原酶方案	*P*1值	*P*2值	*P*3值	无胶原酶方案	含Ⅱ型胶原酶方案	含D型胶原酶方案	*P*1值	*P*2值	*P*3值
CD86	1 169.00±167.40	1 204.00±52.19	1 317.00±212.50	0.9228	0.2714	0.4553	1 221.00±68.25	1 752.00±152.40	1 187.00±71.75	<0.0001	0.8423	<0.0001
CD206	44.68±29.70	149.30±37.02	−56.84±67.22	0.0002	0.0003	<0.0001	21.94±13.48	230.00±15.80	51.77±16.23	<0.0001	0.0105	<0.0001
MHC-Ⅱ	7 007.00±934.40	7 290.00±1249.00	6 892.00±831.90	0.8817	0.9793	0.7814	3 904.00±469.30	4 410.00±758.50	4 377.00±1038.00	0.5231	0.5652	0.9972

**注** GVHD：移植物抗宿主病；*P*1：无胶原酶方案对含Ⅱ型胶原酶方案；*P*2：无胶原酶方案对含D型胶原酶方案；*P*3：含Ⅱ型胶原酶方案对含D型胶原酶方案

## 讨论

cGVHD是allo-HSCT后非复发死亡的主要原因[22]。口腔是cGVHD的主要靶器官，唾液腺功能障碍是口腔cGVHD的主要表现[23]。分离组织单个核细胞进行流式细胞术、质谱流式细胞术和单细胞RNA测序等手段为研究cGVHD靶器官免疫微环境的复杂性提供了新机遇。但是组织消化的方式会不同程度地影响细胞活性及表型，可能无法获得客观的结果。本研究基于动物模型，系统比较3种消化方案对唾液腺免疫细胞表型的影响。

本研究成功构建MHC不相合cGVHD小鼠模型，在移植后第45天收集唾液腺进行组织病理学及流式细胞术分析。与无GVHD组相比，HE染色提示cGVHD组的唾液腺组织出现明显的腺泡萎缩、结构破坏和免疫细胞的浸润，与既往研究一致[10]。Masson三色染色提示cGVHD组出现明显的纤维化，与既往研究一致[8]。使用胶原酶消化在两组中均极大地增加了最终获得的CD45^+^免疫细胞的细胞活率和数量，与GVHD严重程度无关。因此我们认为使用胶原酶消化有助于获取唾液腺的单细胞悬液，从而进行下游的流式细胞术、质谱流式细胞术或者单细胞RNA测序等实验。

本研究结果表明用含胶原酶的消化方案可增加最终获得的CD8^+^T细胞的数量，而含Ⅱ型胶原酶方案处理后获得的CD4^+^T细胞减少，尤其是在cGVHD组中；相比之下，含D型胶原酶方案与无胶原酶方案相比处理后增加CD4^+^ T细胞数量。与既往研究一致，对于CD8^+^T细胞的研究首选Ⅱ型胶原酶[16]，而对于CD4^+^T细胞的研究首选D型胶原酶[17]。本研究结果提示，cGVHD与NK细胞重建失败有关，与既往研究一致[24]。在既往针对其他唾液腺疾病的研究中，分析NK细胞时多采用D型胶原酶进行组织消化[18]。而本研究结果显示，胶原酶处理并不明显影响NK细胞的相对比例，但Ⅱ型胶原酶可获得更多的细胞总量，提示其在唾液腺组织消化效率方面具有优势。此外，本研究结果提示移植后唾液腺中B细胞含量极低，这一特征与其他唾液腺疾病存在明显差异[25]。

中性粒细胞在移植后早期快速向肠道组织迁移，其在肠道中的浸润与aGVHD严重程度密切相关[26]–[27]。我们在cGVHD唾液腺中观察到Ly6C^low^CD11b⁺Ly6G⁺中性粒细胞显著富集。但经Ⅱ型胶原酶处理后几乎无法检测到该中性粒细胞群，而D型胶原酶则显著增加了其检出数量。这提示中性粒细胞对酶消化条件高度敏感，导致其在流式细胞术分析中被系统性低估。此外，我们还鉴定了CD11b^+^Ly6C^+^Ly6G^-^单核细胞群体。在cGVHD背景下，单核细胞的比例和数量与是否使用胶原酶无明显相关性，但D型胶原酶处理始终优于Ⅱ型胶原酶，进一步表明不同胶原酶在保持髓系细胞完整性方面存在差异。

本研究进一步聚焦于CD11c⁺的巨噬细胞。既往研究已明确巨噬细胞是皮肤和肺cGVHD的重要致病细胞[6]。本研究结果提示Ⅱ型胶原酶在回收巨噬细胞方面具有明显优势，且在无GVHD组中更好地保留了其比例特征，这与既往唾液腺疾病中采用Ⅱ型胶原酶研究巨噬细胞的策略一致[15]–[16]。在表型分析中，含Ⅱ型胶原酶方案处理表现出更高的CD86与CD206的MFI值，提示其在保留巨噬细胞表面分子表达完整性方面更具优势。而两种胶原酶对MHC-Ⅱ的表达水平无明显影响。需要指出的是，上述差异同样可能部分来源于不同消化条件对表面抗原检测敏感性的影响，无法完全代表巨噬细胞真实功能状态的改变。

尽管我们对巨噬细胞进行了基于CD86和CD206的初步表型分析，但随着多组学技术的应用，巨噬细胞在cGVHD中的功能异质性和谱系来源已被证明远超传统M1/M2分类框架[28]。因此，本研究的结果更应被视为对样本制备和检测策略的技术性评估。本研究具有以下局限：①我们仅在最常用的MHC不相合cGVHD模型中进行了研究，半相合等其他cGVHD模型中是否存在一样的现象未知。②仅对最常用的2种（Ⅱ型和D型）胶原酶进行了比较，对于其他消化酶的作用还不明确。③仅对主要的免疫细胞类型进行研究，未对比例较少的细胞群，如γ/δT细胞，嗜酸/嗜碱性粒细胞等进行研究。

综上所述，我们通过动物模型对移植后发生cGVHD的唾液腺的消化方案进行探索，明确了不同胶原酶消化对免疫细胞亚群的影响。Ⅱ型胶原酶在分离总免疫细胞、NK细胞、CD8^+^T细胞和巨噬细胞具有优越性；D型胶原酶则有助于富集CD4^+^T细胞、中性粒细胞和单核细胞。本研究从方法学角度提示，不同组织消化方案可显著影响免疫表型检测结果，这一发现对于解释不同研究之间的差异具有重要启示意义。值得注意的是，当细胞膜表面分子为跨膜或膜外结构域较长的表面蛋白，其抗体识别表位极有可能在组织消化过程中受到胶原酶或其他蛋白酶的部分切割或空间遮蔽，从而影响流式细胞术的检测效率。因而，本研究中观察到的表面抗原信号差异，需结合消化条件加以解读，而不应简单等同于真实的生物学表达变化。该方法学结论不仅为cGVHD唾液腺组织免疫学研究提供了重要的方法学依据，对于唾液腺炎症及自身免疫性疾病等研究中优化样本制备方案亦具有潜在参考价值。
